# Improving prediction of heterodimeric protein complexes using combination with pairwise kernel

**DOI:** 10.1186/s12859-018-2017-5

**Published:** 2018-02-19

**Authors:** Peiying Ruan, Morihiro Hayashida, Tatsuya Akutsu, Jean-Philippe Vert

**Affiliations:** 1 Artificial Intelligence Research Center, National Institute of Advanced Industrial Science and Technology (AIST), Tokyo, Japan; 2 Department of Electrical Engineering and Computer Science, National Institute of Technology, Matsue College, 14-4, Nishiikumacho, Matsue, 690-8518 Japan; 3 Bioinformatics Center, Institute for Chemical Research, Kyoto University, Gokasho, Uji, Kyoto, 6110011 Japan; 4 MINES ParisTech, PSL Research University, CBIO-Centre for Computational Biology, Paris, 75006 France; 5 Institut Curie, Paris, 75005 France; 6 INSERM U900, Paris, 75005 France; 7 Ecole Normale Supérieure, Department of Mathematics and Applications, Paris, 75005 France

**Keywords:** Heterodimeric protein complex, Combination kernel, Pairwise kernel

## Abstract

**Background:**

Since many proteins become functional only after they interact with their partner proteins and form protein complexes, it is essential to identify the sets of proteins that form complexes. Therefore, several computational methods have been proposed to predict complexes from the topology and structure of experimental protein-protein interaction (PPI) network. These methods work well to predict complexes involving at least three proteins, but generally fail at identifying complexes involving only two different proteins, called heterodimeric complexes or heterodimers. There is however an urgent need for efficient methods to predict heterodimers, since the majority of known protein complexes are precisely heterodimers.

**Results:**

In this paper, we use three promising kernel functions, Min kernel and two pairwise kernels, which are Metric Learning Pairwise Kernel (MLPK) and Tensor Product Pairwise Kernel (TPPK). We also consider the normalization forms of Min kernel. Then, we combine Min kernel or its normalization form and one of the pairwise kernels by plugging. We applied kernels based on PPI, domain, phylogenetic profile, and subcellular localization properties to predicting heterodimers. Then, we evaluate our method by employing C-Support Vector Classification (C-SVC), carrying out 10-fold cross-validation, and calculating the average F-measures. The results suggest that the combination of normalized-Min-kernel and MLPK leads to the best F-measure and improved the performance of our previous work, which had been the best existing method so far.

**Conclusions:**

We propose new methods to predict heterodimers, using a machine learning-based approach. We train a support vector machine (SVM) to discriminate interacting vs non-interacting protein pairs, based on informations extracted from PPI, domain, phylogenetic profiles and subcellular localization. We evaluate in detail new kernel functions to encode these data, and report prediction performance that outperforms the state-of-the-art.

## Background

Many proteins carry out their biological functions by interacting with other proteins to form multiprotein structures, called protein complexes [1], which are crucial for a broad range of the biological process. For example, the ribosome is an assembly of protein and RNA subunits responsible for protein translation. Therefore, understanding protein functions, as well as biological processes, requires identification of sets of proteins that form complexes. A significant fraction of known protein complexes are heterodimeric protein complexes (heterodimers), that is, formed by the assembly of two different proteins. For example, the two most important protein complex catalogs CYC2008 [2] and MIPS [3] include respectively 172 (42%) and 64 (29%) heterodimers. Hence, it is necessary to develop accurate methods for predicting heterodimers. Here CYC2008 is a comprehensive catalog of 408 manually curated yeast protein complexes reliably supported by small-scale experiments, and MIPS provides detailed information involving classification schemes for analysis of protein sequences, RNA genes, and other genetic elements [4–6].

Several high-throughput methods have supplied us with large datasets of protein-protein interactions (PPIs) [7, 8], such as tandem affinity purification (TAP) and yeast two-hybrid (Y2H) [9]. To predict protein complexes, many researchers have proposed to study the structure of the resulting PPI network [10–15], which is an undirected graph with proteins represented as vertices and interactions between them represented as edges. For example, methods such as Markov Cluster (MCL) [16], Molecular Complex Detection (MCODE) [17], Clustering-based on Maximal Cliques (CMC) [18], Protein Complex Prediction (PCP) [19], and CFinder [20] are mainly based on the topological structures of PPI networks. Other methods such as Restricted Neighborhood Search Clustering (RNSC) [21] and Feng et al. [22] exploit biological information such as microarray data and gene ontology (GO) to strengthen the reliability of interactions so as to rebuild a more reliable PPI network and to predict complexes through a subgraph detection method from such PPI network. Some supervised approaches such as Bayesian classifier [23] also have been proposed. These methods, however, focus mainly on detecting densely connected subgraphs in PPI networks and are therefore not adapted to the identification of heterodimers. Indeed, for a complex involving only two proteins, the structure of the PPI network restricted to the involved two proteins is reduced to the presence or absence of an edge between them, and the prediction boils down to experimentally measured interaction. The methods above are not satisfactory because (i) high-throughput experimental measures are known to have high rates of false positives and false negatives, and (ii) two interacting proteins do not necessarily form a heterodimer, as they may instead be involved in a larger complex. As a result, it is difficult to predict heterodimers accurately with these methods, which have been evaluated for their ability to predict protein complexes consisting of at least three proteins.

Another class of methods focuses specifically on the prediction of heterodimers, using either random walks on PPI networks, such as the Repeated Random Walks (RRW) method [24] and the Node-Weighted Expansion (NWE) method [25], or a naive Bayes classifier as proposed by Maruyama [26], with features combining PPI data, GO annotations, and gene expression data. The later method has been shown to have better performance in F-measure for prediction of heterodimers than other existing prediction methods, including MCL, MCODE, RRW, and NWE.

To improve the prediction accuracy for heterodimers, Ruan et al. [27] proposed a supervised method with several features based on PPI weights. The weights are obtained from dataset WI-PHI (a Weighted yeast Interactive enriched for direct PHysical Interactions), which includes 49607 interacting protein pairs except self interactions and the weights of interactions between protein pairs. The main idea behind the design of feature space mappings is that the neighboring weights of a heterodimer tend to be smaller than the weight inside of the heterodimer. In addition to features based on weights, they proposed feature space mappings based on the number of protein domains because domains are considered to be functional and structural units in proteins. Furthermore, they designed a Domain Composition kernel based on the idea that two proteins having the same composition of domains as a known heterodimer are likely to form a heterodimer. The method showed considerable promise for heterodimer detection (F-measure=63.1%), significantly outperforming previous works.

Yong et al. [28] proposed a two-stage approach and test their approach on the prediction of yeast and human small complexes (consisting two or three distinct proteins). They carried out comparison with some popular complex prediction methods. Besides, they generated a larger number of novel predictions. However, on prediction of yeast heterodimers, they did not provide the measure performances of precision and recall. Therefore, we have no idea whether or not they achieve better performance than Ruan et al. [27] based on their results.

Note that Yugandhar et al. [29] applied a machine learning approach to classify protein-protein complexes based on their binding affinities. Their method reaches 76.1% accuracy to distinguish heterodimers into high and low affinity groups. However, they classify known heterodimers into different groups, but do not predict heterodimers from given protein pairs, hence their purpose is different from ours.

In this paper, our goal is to further improve the prediction accuracy for heterodimers. We investigate combination kernels to encode the domain composition of proteins involved in a complex since the one used in Ruan et al. [27] was very crude. More precisely, they define the similarity of domain composition in protein pairs very strictly, only considering two protein pairs with exactly the same compositions as an effective feature in the kernel function. We find that there is space to improve prediction from this point by replacing “exactly the same” with “similar”. For that purpose we propose to replace the Dirac kernel (which is 1 if and only if two proteins have exactly the same domain composition, 0 otherwise) by the so-called Min kernel, which counts the number of shared domains between two proteins. Furthermore, since our problem is formally to classify *pairs* of proteins as interacting or not, we exploit the notion of *pairwise* kernels to extend kernels between individual proteins to kernels between pairs of proteins, investigating in particular the *metric learning pairwise kernel* (MLPK) and *tensor product pairwise kernel* (TPPK), as explained in [30] and in the “Methods” section.

Besides, we consider that various sources of information may contribute to an accurate predictor. The combination of various sources can be divided into three situations: (1)various types of features with a single kernel; (2)one type of features with multiple kernels; (3)various types of features with multiple kernels. We test all the three situations and show only significant results in our computational experiments. On various types of features, besides the protein-protein interaction (PPI) and domain properties, we also try to use phylogenetic profile property. The reason is that two proteins that are both present or absent in the same genome are likely to have related functions. Moreover, protein subcellular localization property is considered as well. As proteins must be localized at their appropriate subcellular compartment to perform their function, proteins in the same location may have similar functions. On multiple kernels, we employ Min kernel and its two normalization forms, MinMax kernel and Scaled Min kernel, as well as two pairwise kernels, MLPK and TPPK.

Then, we employ C-Support Vector Classification (C-SVC), carry out ten-fold cross-validation and calculate the average precision, recall, and F-measures. The computational experiments show that using Min kernel improves the prediction performance, and the combinations of multiple kernels outperform single Min kernel, therefore is superior to [27] and other existing methods. However, combinations of new types of features that we presented do not contribute to accuracy improvement. Thus, situation (2) is more appropriate to our problem, though we do not eliminate the effectiveness of situation (3) by adding other useful types of features.

The rest of paper is organized as follows: “Methods” section introduces our methods including details of kernel combination and other types of features. “Results” section presents performance evaluation and comparison with other methods, as well as discussion on the results. “Discussion” section concludes the paper.

## Methods

We formulate the problem of heterodimer prediction as a supervised binary classification problem. Given a set of pairs of proteins that known to form heterodimers (positive examples), and pairs of proteins that do not form heterodimers (negative examples) as training data, we learn a function *f*(*x*) to predict if a pair *x* of proteins in the test set can form a heterodimer (*f*(*x*)≥0) or not (*f*(*x*)<0). The definition of positive examples and negative examples are the same as [27]. To learn the function *f*(*x*) from a training set (*x*_1_,*y*_1_),…,(*x*_*n*_,*y*_*n*_), where each $x_{i} \in \mathbb {R}^{p}$ is a vector of descriptors for a pair of proteins and *y*_*i*_∈{−1,1} indicates whether the pair can form a complex or not, we employ a *C*-support vector classification (*C*-SVC) classifier, with balanced loss penalty to compensate for the fact that the numbers of positive examples and negative examples are very unbalanced.

### Various properties and multiple kernels

We explain multiple kernels involving properties of PPI, domain, phylogenetic profile, and subcellular localization in this section.

#### PPI and domain properties

For the PPI and domain properties, we follow the work in [27], feature space mapping ***ψ*** for a pair of proteins *P*_*i*_, *P*_*j*_ is defined as

1 $$ {\begin{aligned} \boldsymbol{\psi}(P_{i}, P_{j})=\left(\begin{array}{c} w_{ij} \\ \max \left\{\max_{\{k|(i,k)\in E, k\neq j\} }w_{ik}, \max_{\{k|(j,k)\in E, k\neq i\}}w_{jk}\right\} \\ \min \left\{\min_{\{k|(i,k)\in E, k\neq j\} }w_{ik}, \min_{\{k|(j,k)\in E, k\neq i\}}w_{jk}\right\} \\ \max_{\{k|(i,k)\in E, (j,k)\in E\}} \min\{w_{ik}, w_{jk}\} \\ \max_{\{k_{1},k_{2}|(i, k_{1})\in E, k_{1} \neq j, (j, k_{2})\in E, k_{2} \neq i\}}\left\vert\, w_{{ik}_{1}}-w_{{jk}_{2}} \,\right\vert\\ \max\{\# \text{domains of} P_{i}, \# \text{domains of}~ P_{j}\}\\ \min\{\# \text{domains of} P_{i}, \# \text{domains of}~ P_{j}\}\ \end{array}\right), \end{aligned}}  $$

where *w*_*ij*_ denotes the weight of the interaction between *P*_*i*_ and *P*_*j*_. These are novel features proposed by Ruan et al., and the detailed descriptions of each feature can be found in [27].

There is another method involving domain property proposed in [27], called Domain Composition kernel. Here we briefly review it, since our approach is mainly on improving this part.

**Fig. 1:**
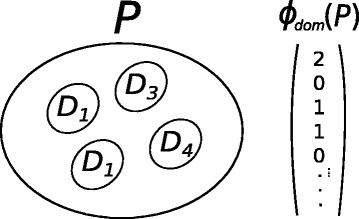
Illustration for ***ϕ***_***dom***_(*P*_*i*_). Left: a protein *P*_*i*_ includes domains *D*_1_, *D*_1_, *D*_3_ and *D*_4_. Right: the corresponding feature space mapping ***ϕ***_*dom*_ for protein *P*_*i*_

Suppose that there are several domains *D*_*j*_ in proteins. We define a feature space mapping ***ϕ***_***dom***_ for protein *P*_*i*_ so that the *j*-th element of ***ϕ***_***dom***_(*P*_*i*_) is the number of domains of *D*_*j*_ in *P*. For example, in Fig. 1, the left side is a protein *P*_*i*_ with domains *D*_1_,*D*_1_,*D*_3_,*D*_4_ and the right side is the corresponding feature space mapping ***ϕ***_***dom***_(*P*_*i*_) with values (2,0,1,1,0,⋯) representing 2 *D*_1_*s*, 0 *D*_2_, 1 *D*_3_, 1 *D*_4_, 0 *D*_5_, and so on, included in protein *P*_*i*_. The dimension of ***ϕ***_***dom***_(*P*_*i*_) is the total number of distinct domains contained in the whole proteins.

The formulation of Domain Composition kernel *K*_*C*_ for two pairs of proteins, (*P*_1_,*P*_2_) and (*P*_3_,*P*_4_), is defined as

2 $$ {{}\begin{aligned} K_{C}((P_{1}, P_{2}), (P_{3}, P_{4}))&=\max\{\delta(\boldsymbol{\phi_{dom}}(P_{1})=\boldsymbol{\phi_{dom}}(P_{3}))\\&\quad\delta(\boldsymbol{\phi_{dom}}(P_{2})=\boldsymbol{\phi_{dom}}(P_{4})),\\ \delta(\boldsymbol{\phi_{dom}}(P_{1})&=\boldsymbol{\phi_{dom}}(P_{4}))\\&\quad\delta(\boldsymbol{\phi_{dom}}(P_{2})=\boldsymbol{\phi_{dom}}(P_{3}))\}, \end{aligned}}  $$

where *δ*(*S*)=1 if *S* holds, otherwise 0. It should be noted that the Domain Composition kernel is actually defined for pairs of two or more proteins.

In this study, we focus on replacing Domain Composition kernel with more promising combination kernels. Before presenting combination kernels, we first continuously introduce other properties.

#### Phylogenetic profile property

The phylogenetic profile of a protein is a vector that describes the presence or absence of homologs in organisms. It has been studied that proteins having similar profiles strongly tend to be functionally linked [31], and it is well known that proteins with similar functions are likely to form a complex. Therefore, we consider that phylogenetic profiles may be helpful for determining heterodimers.

To represent the subset of organisms that contain a homolog, we constructed a phylogenetic profile for each protein. This profile is a vector with *m* entries, where *m* corresponds to the number of genomes (2, 717 in the present article). We indicate the presence of a homolog to a given protein in the *j*-th genome with an entry of unity at the *j*-th element. If no homolog is found, the element is zero.

We compute phylogenetic profiles for the 5, 497 proteins encoded by the genome from KEGG OC [32], a novel database of ortholog clusters. Each protein sequence (*P*_*i*_) is encoded by 2, 717 genomes, which consist of eukaryotes, bacteria and archaea. Proteins coded by the *j*-th genome are defined as including a homolog of a protein *P*_*i*_ if they align to the protein *P*_*i*_ with a score that is deemed statistically significant.

**Fig. 2:**
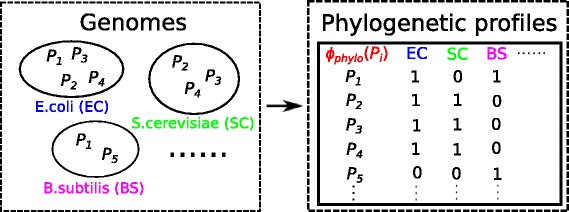
Illustration for ***ϕ***_***phylo***_(*P*_*i*_). Left: Genomes with their proteins. For example, *EC* contains *P*_1_, *P*_2_, *P*_3_ and *P*_4_, *SC* contains *P*_2_, *P*_3_ and *P*_4_, *BS* contains *P*_1_ and *P*_5_. Right: Phylogenetic profile *ϕ*_*phylo*_(*P*_*i*_) for each protein *P*_*i*_

In Fig. 2, the left side are several genomes with their proteins and the right side are phylogenetic profiles for all proteins. We define a feature space mapping ***ϕ***_***phylo***_ for protein *P*_*i*_ so that the *j*-th element of ***ϕ***_***phylo***_(*P*_*i*_) describes whether or not the *j*-th genome contains *P*_*i*_. For example, in the genomes, *P*_1_ exists in *EC* and *BS* but not in *SC*, so for the phylogenetic profile of protein *P*_1_, elements of *EC* and *BS* are 1, and *SC* is 0.

#### Subcellular localization property

**Fig. 3:**
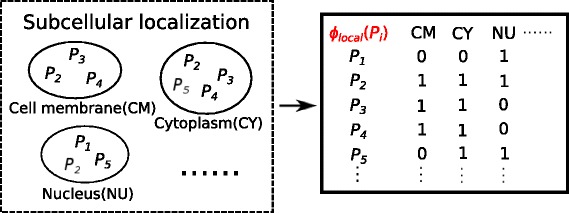
Illustration for ***ϕ***_***local***_(*P*_*i*_). Left: Proteins contained in each subcellular localization. For example, in cell membrane, there are proteins *P*_2_, *P*_3_ and *P*_4_ inside; in cytoplasm,there are *P*_2_, *P*_3_, *P*_4_ and *P*_5_; in nucleus, there are *P*_1_, *P*_2_ and *P*_5_ contained. Right: A feature space mapping *ϕ*_*local*_(*P*_*i*_) of localization information for each protein *P*_*i*_

Determining the subcellular localization of a protein is a key step toward understanding the cellular function of a protein, since proteins of the same subcellular localization tend to have similar function. We obtain the subcellular localization information for each protein from UniProtKB, such as cell membrane, cytoplasm, nucleus, and so on. Similar with phylogenetic profile property, we construct a feature space mapping ***ϕ***_***local***_(*P*_*i*_) containing subcellular localization information for each protein *P*_*i*_. The size of feature space is the sum of unique localizations for all proteins in our experiments, with elements 1 and 0, each represents whether or not the corresponding protein exists in the location (shown as Fig. 3).

#### Multiple kernels

In this section, we start to describe Min kernel with its normalization forms and two pairwise kernels.

Min kernel [33] counts the number of common elements in two feature vectors, which is a simple way to calculate the similarity of two binary vectors. Different from Domain Composition kernel, which outputs 1 or 0 representing exactly the same or not two protein pairs are, Min kernel counts the number of common domains in two proteins. With combining pairwise kernel presented below, combined-Min kernel shows the similarity of domain composition between protein pairs. Note that Min kernel has been shown to be useful for detection and recognition in [34, 35]. For feature vectors ***x***,***y***, the Min kernel *K*_*Min*_ is defined by

3 $$  K_{Min}(\boldsymbol{x, y})=\sum\limits_{i=1}^{n}\min\{x_{i},y_{i}\},  $$

where *x*_*i*_ denotes *i*-th element of vector ***x***, *n* denotes the number of elements of ***x***, and *x*_*i*_,*y*_*i*_≥0 for all *i*.

When we present a kernel, its normalization form is usually used in kernel functions to improve prediction accuracy. Therefore, normalized versions are also proposed. Scale-normalization is a very common normalized version. For some kernel *K*, a scale-normalized kernel is defined as

4 $$  K_{norm}(\boldsymbol{x}, \boldsymbol{y})=\frac{K(\boldsymbol{x}, \boldsymbol{y})}{\sqrt{K(\boldsymbol{x}, \boldsymbol{x})K(\boldsymbol{y},\boldsymbol{y})}}.  $$

Tanimoto kernel has been shown to have good performance on pairwise problems in the previous study [36], and it has a simple expression when applying to the Min kernel, which is called MinMax kernel. As a result, MinMax kernel is regarded as another normalization form of Min kernel. It computes the ratio of the intersection to the union of two feature mappings. For feature vectors ***x***,***y***, MinMax kernel *K*_*MinMax*_ is defined as

5 $$  K_{MinMax}(\boldsymbol{x},\boldsymbol{y})=\frac{K_{Min}(\boldsymbol{x},\boldsymbol{y})}{{\sum\nolimits}_{i=1}^{n}\max\{x_{i},y_{i}\}},  $$

where *K*_*Min*_ is Min kernel.

Next, we briefly review two pairwise kernels, the Metric Learning Pairwise Kernel (MLPK) [30] and Tensor Product Pairwise Kernel (TPPK) [37].

Vert et al. [30] presents that MLPK kernel is a kernel for pairs and can be easily used to solve supervised classification problems. For heterodimer prediction problem, it infers pairwise relationships from hetero-protein pairs by defining a kernel between pairs of proteins from a kernel between individual proteins. MLPK kernel *K*_*MLPK*_ between pairs (***x***_1_,***x***_2_) and (***x***_3_,***x***_4_) is defined as

6 $$ \begin{aligned} K_{MLPK}((\boldsymbol{x}_{1}, \boldsymbol{x}_{2}),(\boldsymbol{x}_{3}, \boldsymbol{x}_{4})) &= (K(\boldsymbol{x}_{1}, \boldsymbol{x}_{3})-K(\boldsymbol{x}_{1}, \boldsymbol{x}_{4})\\& \quad-K(\boldsymbol{x}_{2}, \boldsymbol{x}_{3})+K(\boldsymbol{x}_{2}, \boldsymbol{x}_{4}))^{2}, \end{aligned}  $$

The rationale behind MLPK is that the comparison between a pair (***x***_1_,***x***_2_) and another pair (***x***_3_,***x***_4_) is done through comparing the feature space of pair *K*(***x***_1_,***x***_3_)+*K*(***x***_2_,***x***_4_) and that of pair *K*(***x***_1_,***x***_4_)+*K*(***x***_2_,***x***_3_). In other words, MLPK compares pairs through the differences between their elements in the feature space.

Different from MLPK, TPPK kernel compares pairs by comparing ***x***_1_ with ***x***_3_ and ***x***_2_ with ***x***_4_ on one hand, and comparing ***x***_1_ with ***x***_4_ and ***x***_2_ with ***x***_3_ on the other. Both comparisons are obtained by a tensorization of the initial feature space. Therefore, this pairwise kernel is called the tensor product pairwise kernel. The equation of TPPK kernel is defined as

7 $$ \begin{aligned} K_{TPPK}((\boldsymbol{x}_{1}, \boldsymbol{x}_{2}),(\boldsymbol{x}_{3}, \boldsymbol{x}_{4})) &= K(\boldsymbol{x}_{1}, \boldsymbol{x}_{3})K(\boldsymbol{x}_{2}, \boldsymbol{x}_{4})\\& \quad+ K(\boldsymbol{x}_{1}, \boldsymbol{x}_{4})K(\boldsymbol{x}_{2}, \boldsymbol{x}_{3}), \end{aligned}  $$

### Kernel combinations

So far, we have mentioned three kernels between proteins: Min kernel, and two normalized versions, MinMax kernel and scaled kernel (called Normalized kernel in the results), as well as two pairwise kernels between protein pairs, MLPK kernel and TPPK kernel. We therefore consider all possible combinations (3×2=6) of these kernels.

For two protein pairs (*P*_1_, *P*_2_) and (*P*_3_, *P*_4_), we have the following combinations.

8 $$ {\begin{aligned} K_{M}((P_{1},P_{2}),(P_{3},P_{4})) &= (K(\boldsymbol{\phi}(P_{1}),\boldsymbol{\phi}(P_{3})) - K(\boldsymbol{\phi}(P_{1}),\boldsymbol{\phi}(P_{4}))\\ &\quad- K(\boldsymbol{\phi}(P_{2}),\boldsymbol{\phi}(P_{3})) \,+\, K(\boldsymbol{\phi}(P_{2}),\boldsymbol{\phi}(P_{4})))^{2}, \end{aligned}}  $$

9 $$ {\begin{aligned} K_{T}((P_{1},P_{2}),(P_{3},P_{4})) &= K(\boldsymbol{\phi}(P_{1}), \boldsymbol{\phi}(P_{3})) K(\boldsymbol{\phi}(P_{2}),\boldsymbol{\phi}(P_{4}))\\ &\quad+ K(\boldsymbol{\phi}(P_{2}),\boldsymbol{\phi}(P_{3}))K(\boldsymbol{\phi}(P_{1}), \boldsymbol{\phi}(P_{4})), \end{aligned}}  $$

where *K*(***ϕ***(*P*_*i*_),***ϕ***(*P*_*j*_)) denotes Min kernel or one of its normalized versions in the two equations. That is to say, we plug Min kernel and its normalized versions into Eqs. (8) and (9), respectively. Note that ***ϕ***(*P*_*i*_) can be any one of ***ϕ***_***dom***_(*P*_*i*_), ***ϕ***_***phylo***_(*P*_*i*_) and ***ϕ***_***local***_(*P*_*i*_).

Then we combine the feature space mapping ***ψ*** (Eq. (1)) with the 6 combinations above, so we have

10 $$\begin{array}{@{}rcl@{}} {} K_{comb}((P_{1},P_{2}),(P_{3},P_{4}))&=&\langle\boldsymbol{\psi}(P_{1}, P_{2}), \boldsymbol{\psi}(P_{3}, P_{4})\rangle \\ &&+ \alpha K((P_{1},P_{2}),(P_{3},P_{4})), \end{array} $$

where *α* is a constant, and *K* is either of the 6 combination kernels. We call *K*_*comb*_ using *K*_*Min*_ “Min-MLPK kernel”, using *K*_*MinMax*_ “MinMax-MLPK kernel”, using *K*_*norm*_ “Normalized Min-MLPK kernel”, respectively. Similarly, when applying TPPK kernel, we just need to replace “MLPK” with “TPPK” for their names.

The study [30] pointed out that combination of MLPK and TPPK together by summation almost always leads to the best results. Therefore, by summation with MLPK (Eq. (8)) and TPPK equation (Eq. (9)), we have

11 $$\begin{array}{@{}rcl@{}} {} K_{comb}((P_{1},P_{2}),(P_{3},P_{4}))&=&\langle\boldsymbol{\psi}(P_{1}, P_{2}), \boldsymbol{\psi}(P_{3}, P_{4})\rangle  \\ &&+ \alpha K_{M}((P_{1},P_{2}),(P_{3},P_{4}))  \\ &&+ \alpha K_{T}((P_{1},P_{2}),(P_{3},P_{4})),\,\,\, \end{array} $$

We call *K*_*comb*_ using *K*_*M*_ and *K*_*T*_ “MinMax-MLPK-TPPK kernel”.

### *C*-Support Vector Classification(*C*-SVC)

We use the *C*-Support Vector Classification (*C*-SVC) [38, 39] formulation that infers a function *f*(***x***)=***w***^⊤^***x*** that best separates positive examples from negative ones by solving the optimization problem:

12 $$ \begin{aligned} & \text{minimize} & & \frac{1}{2} \|\boldsymbol{w}\|^{2}+C^{+}\sum\limits_{y_{i}=+1}{\xi_{i}}+C^{-}\sum\limits_{y_{i}=-1}{\xi_{i}}\\ & \text{subject to} & & y_{i}\left(\boldsymbol{w}^{\top} \boldsymbol{x_{i}} +b\right) \geq 1 - \xi_{i}, \text{for all}\ {i}\\ & & & \xi_{i} \geq 0, \text{for all} {i} \end{aligned}  $$

where *C*^+^ and *C*^−^ are regularization parameters for positive and negative examples, respectively. Instead of representing explicitly each pair of proteins by a vector of descriptors ***x***∈*R*^*p*^, we will use positive definite kernels *K*(***x***,***x***^***′***^) in which case the *C*-SVC classifier takes the form $f(\boldsymbol {x}) = {\sum \nolimits }_{i=1}^{n} \alpha _{i} K(\boldsymbol {x_{i}},\boldsymbol {x})$ where the vector ***α***∈*R*^*n*^ is the solution of the dual problem:

13 $$ \begin{aligned} & \text{minimize} & & \boldsymbol{\alpha}^{\top} \mathbf{K} \boldsymbol{\alpha} - 2 \boldsymbol{\alpha}^{\top} \mathbf{1}\\ & \text{subject to} & & 0 \leq \alpha_{i} \leq C^{+}, \text{if }y_{i}=1\\ & & & 0 \leq -\alpha_{i} \leq C^{-}, \text{if }y_{i}=-1\\ \end{aligned}  $$

where **K** is the *n*×*n* Gram matrix with entries **K**_*ij*_=*K*(***x***_***i***_,***x***_***j***_) and **1** is the *n*-dimensional vector of ones. For implementation of *C*-SVC, we used *libsvm* (version 3.11) [40].

## Results

### Experiments

In order to compare our proposed method with the method in [27], we used the same dataset WI-PHI. The weights of interactions were calculated in the following way. (1)Used the high-throughput yeast two-hybrid data by Ito [8] and Uetz [7] as well as several databases such as BioGRID [11], MINT [12] and BIND [13] to build the literature-curated physical interaction (LCPH) dataset. (2)Constructed a benchmark dataset to evaluate high-throughput data. The interactions of the dataset were obtained by two independent methods from LCPH-LS, which was a low-throughput dataset in LCPH. (3)Calculated a log-likelihood score (LLS) to each dataset except LCPH-LS. (4)Computed the weight of each interaction by multiplying the socioaffinity (SA) indices [1] and the LLSs from different datasets. Note that SA index is the log-odds score of the number of times that we observed two proteins interact to each other to the expected value in the dataset.

Also, we prepared the same dataset from CYC2008 [2] for training and testing as the previous study. CYC2008 is a set of 408 manually curated yeast complexes. Compared with MIPS catalogue, which consists 215 heteromeric complexes, we believe that CYC2008 represents a more complete and up-to-date description of the stable yeast interactome, and should hence serve as an improved gold standard for the prediction of complexes. CYC2008 catalogue can be downloaded at: http://wodaklab.org/cyc2008/.

We defined a positive example as a pair of proteins included in WI-PHI as well as a heterodimer included in CYC2008. A negative example was defined as a pair of proteins included in WI-PHI, which meanwhile should not be any heterodimer but be a subset of some other complexes in CYC2008. As a result, we had 152 positive examples and 5345 negative examples.

### Performance measure

We chose the following three measures to evaluate our performance. Precision describes the rate of correctly predicted positive examples to all positively predicted examples, and recall describes the rate of correctly predicted positive examples to all positive examples. Both of them indicate the effectiveness of the method from different aspects. F-measure is defined as their harmonic mean, which was used for evaluating the balance of precision and recall since it is insufficient to evaluate by any single one of them.

They are defined as

14 $$ {\kern6pt}\text{precision} = \frac{TP}{TP+FP},  $$

15 $$ {\kern21pt}\text{recall} = \frac{TP}{TP+FN},  $$

16 $$ \text{F-measure} = \frac{2\cdot \text{precision} \times \text{recall}}{\text{precision} + \text{recall}},  $$

where *TP*,*FP*, and *FN* represent the numbers of correctly predicted positive examples, incorrectly predicted positive examples, and incorrectly predicted negative examples, respectively.

### Results

**Fig. 4:**
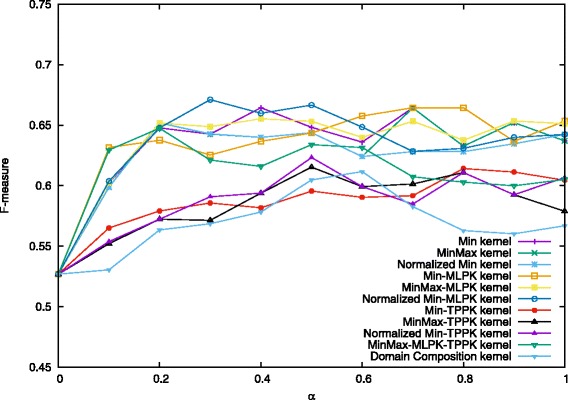
Performance on prediction for heterodimers when *C*^+^=3.5. Values in the figure are average F-measure for each combination kernel method with *α* from 0 to 1.0 when *C*^+^=3.5 and *C*^−^=1.0. The first four kernels are proposed methods and the last one is the existing best method

**Fig. 5:**
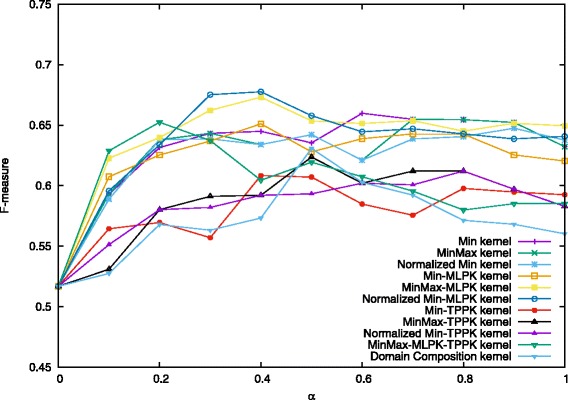
Performance on prediction for heterodimers when *C*^+^=4.0. Values in the figure are average F-measure for each combination kernel method with *α* from 0 to 1.0 when *C*^+^=4.0 and *C*^−^=1.0. The first four kernels are proposed methods and the last one is the existing best method

**Fig. 6:**
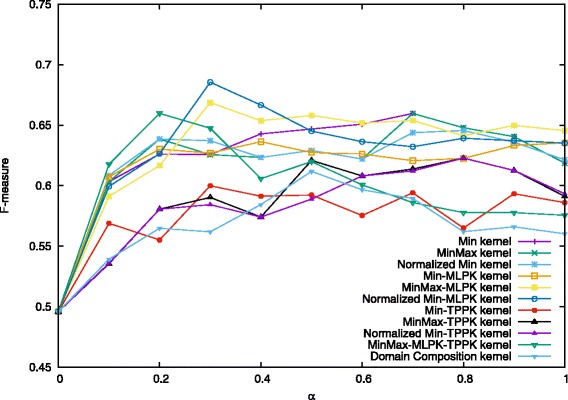
Performance on prediction for heterodimers when *C*^+^=4.5. Values in the figure are average F-measure for each combination kernel method with *α* from 0 to 1.0 when *C*^+^=4.5 and *C*^−^=1.0. The first four kernels are proposed methods and the last one is the existing best method

We present below a comparison of our proposed combination kernels and the best existing method [27], which is titled as “Domain Composition kernel” in Figs. 4, 5 and 6. Note that features shown in Eq. (1) were used both in this study and [27].

In [27], they employed *C*-SVC with varying mixing parameter *α* = 0.0, 0.1, 0.2,..., 2.0. and regularization parameters *C*^−^ = 0.1, 0.2, ⋯, 2.0, *C*^+^ = 3.0, 3.5, ⋯, 6.0. The best result was obtained for *α* = 0.5, *C*^−^ = 1.0 and *C*^+^ = 4.0. In their experiments, they found that the results almost did not change while *C*^−^ varied. Therefore, in this study, we kept the value of *C*^−^ as 1.0, and set other parameters around the best value: *α* = 0.0, 0.1, 0.2,..., 1.0, and *C*^+^ = 3.5, 4.0, 4.5. By performing 10-fold cross-validation each time and taking the average of precision, recall, and F-measure, we used the same experimental procedure to compare the performance of Min kernel and its normalization forms, as well as kernels combining MLPK, TPPK and their summation MLPK + TPPK. The results are shown in Figs. 4, 5 and 6.

When *C*^+^ = 4.5 and *α* = 0.3, Normalized Min-MLPK kernel attains the best F-measure 0.686, compared with 0.631 in [27]. Figure 5 shows the results of each combination kernel on the average F-measures for the case when *C*^+^ = 4.0, *C*^−^ = 1.0 and *α* = 0.0, 0.1, 0.2,..., 1.0. The best two results 0.678 and 0.675 were obtained by the Normalized Min-MLPK kernel when *α* = 0.4 and 0.3, respectively. The third best result 0.673 was obtained by MinMax-MLPK kernel when *α* = 0.4.

These three figures indicate that all the MLPK-combined kernels outperform previously proposed Domain Composition kernel for every value of *α*, as well as all min kernels and its normalization forms, while TPPK-combined kernels are similar with Domain Composition kernel, even a little lower at some points. It demonstrates that converting to MLPK pairwise kernel indeed leads to better prediction performance.

## Discussion

The better performance of MLPK compared to TPPK implies that protein pairs in the training set are similar to other pairs, but not similar to each other. This observation is not surprising because the composition of domains in given protein pairs and known heterodimers (protein pairs) are expected similar, while they do not have to be similar with each other. That is also the reason why Ruan et al. proposed Domain Composition kernel in [27]. It also confirms that the pairwise kernels deduced from the addition of the individual kernels performs better than the addition of the pairwise kernels deduced from individual kernels. Another interesting observation is that, although Vert et al. [30] showed that the summation of MLPK and TPPK almost always led to best results, regarding to our problem, the combination of MLPK and TPPK almost has performance between MLPK and TPPK.

**Fig. 7:**
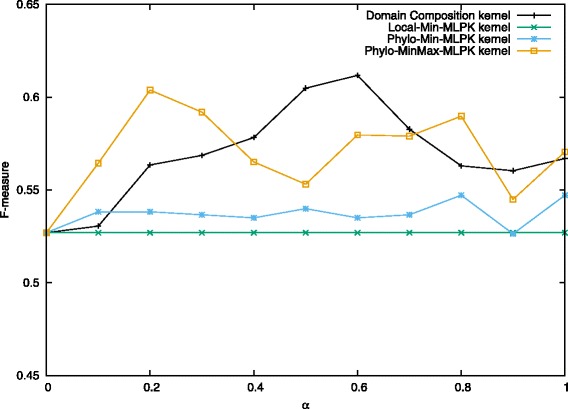
Performance on prediction for heterodimers using phylogenetic and localization information when *C*^+^ = 3.5. Values in the figure are average F-measure for each combination kernel method with *α* from 0 to 1.0 when *C*^+^=3.5 and *C*^−^=1.0. The first one kernel is localization information based Min-MLPK kernel and the left two are phylogenetic information based Min-MLPK kernel and MinMax-MLPK kernel, respectively

**Fig. 8:**
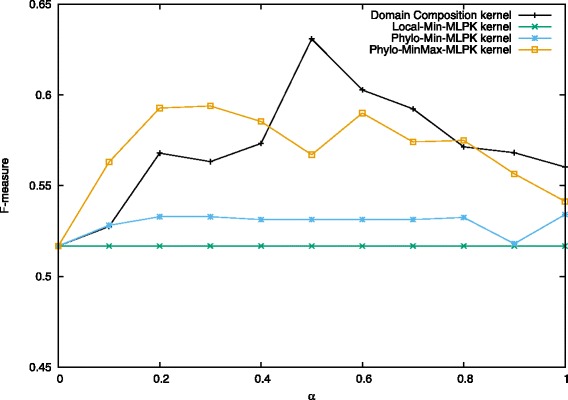
Performance on prediction for heterodimers using phylogenetic and localization information when *C*^+^ = 4.0. Values in the figure are average F-measure for each combination kernel method with *α* from 0 to 1.0 when *C*^+^=4.0 and *C*^−^=1.0. The first one kernel is localization information based Min-MLPK kernel and the left two are phylogenetic information based Min-MLPK kernel and MinMax-MLPK kernel, respectively

**Fig. 9:**
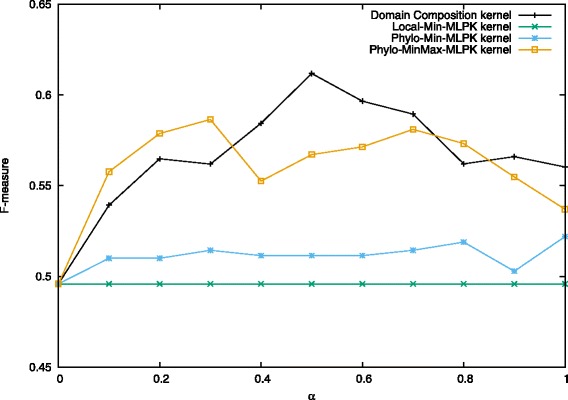
Performance on prediction for heterodimers using phylogenetic and localization information when *C*^+^ = 4.5. Values in the figure are average F-measure for each combination kernel method with *α* from 0 to 1.0 when *C*^+^=4.5 and *C*^−^=1.0. The first one kernel is localization information based Min-MLPK kernel and the left two are phylogenetic information based Min-MLPK kernel and MinMax-MLPK kernel, respectively

We also show the results of subcellular localization property and phylogenetic profile property in Figs. 7, 8, 9. The results of localization keep the same and low as *α* changes. So it suggests that, unfortunately, localization property has no contribution to predicting heterodimers. This is surprising at first since two proteins could form a complex only if they are co-localized. However, the localization data is somewhat not complete because not all of yeast proteins are assigned localization, and many proteins are assigned to multiple locations. As a result, the information turns out to be not useful because only a small part of protein pairs share exactly the same localization.

For phylogenetic profile property, it performs better than [27] at many points when we applied MinMax-MLPK kernel to it, while performing worse when applying Min-MLPK kernel. In addition, we observed that Normalized Min-MLPK kernel and MinMax-MLPK kernel had better performances in most cases. The observation shows that the normalization form has contribution to improving prediction accuracy.

Table 1 shows the exact performance of each combination kernel on their best average precision, recall, and F-measure. Normalized Min-MLPK kernel had the best performance on precision (increased from 61.8 to 71.7%) and MinMax kernel had the best performance on recall (increased from 64.4 to 71.8%). Normalized Min-MLPK kernel achieved the best performance on F-measure (increased from 63.1 to 68.6%) and all the proposed methods that exclude TPPK-combined kernels outperform Domain Composition kernel. The last 5 rows are the results of other existing state-of-the-art methods, which were all given the same dataset WI-PHI as ours and executed with their default settings to predict heterodimers, except the option of the minimum size of predicted complexes, which was set to be two.

**Table 1:** Performance on the best average precision, recall, and F-measure for each combination kernel and other methods

Method	*α*	*C* ^−^	*C*^+^/*C*^−^	Precision	Recall	F-measure
Min kernel	0.7	1.0	3.5	0.658	0.671	0.664
MinMax kernel	0.7	1.0	3.5	0.618	0.718	0.664
Normalized Min kernel	0.2	1.0	3.5	0.678	0.628	0.652
Min-MLPK kernel	0.4	1.0	3.5	0.664	0.664	0.664
MinMax-MLPK kernel	0.4	1.0	4.0	0.678	0.669	0.673
Normalized Min-MLPK kernel	0.3	1.0	4.5	0.717	0.657	0.686
Min-TPPK kernel	0.8	1.0	3.5	0.539	0.713	0.614
MinMax-TPPK kernel	0.5	1.0	4.0	0.605	0.643	0.624
Normalized Min-TPPK kernel	0.5	1.0	4.0	0.605	0.643	0.624
MinMax-MLPK-TPPK kernel	0.2	1.0	4.5	0.632	0.691	0.660
loc-Min-MLPK kernel	0.0	1.0	3.5	0.667	0.506	0.527
phy-Min-MLPK kernel	0.8	1.0	3.5	0.612	0.521	0.547
phy-MinMax-MLPK kernel	0.2	1.0	3.5	0.632	0.578	0.604
Domain Composition kernel [27]	0.5	1.0	4.0	0.618	0.644	0.631
naive Bayes [26]	-			0.24	0. 44	0.31
MCL [16]	-			0.017	0. 023	0.020
MCODE [17]	-			0	0	-
RRW [24]	-			0.030	0.32	0.055
NWE [25]	-			0.035	0.33	0.063

The table lists, for each kernel combination, the average precision, recall, and F-measure are obtained in a 10-fold cross-validation experiment. The results by the naive Bayes-based method [26], MCL [16], MCODE [17], RRW [24], and NWE [25] are also shown, where the experiments for these methods were performed in [26]

## Conclusions

We applied multiple combination kernels based on various types of information, such as protein protein interaction, domain, subcellular localization, and phylogenetic profile to predicting heterodimers. We combined Min kernel (or its normalized forms) with the information above and a pairwise kernel (MLPK or TPPK) by plugging. To evaluate our proposed method, we performed ten-fold cross-validation computational experiments for the combination kernels. The results suggest that our proposed method improved the performance of our previous work, which had been the best existing method so far. In particular, the Normalized Min-MLPK has the best performance.

We indicated that for the problem of predicting heterodimeric protein complexes, multiple combination kernels have better performance than single kernel, and proved that MLPK-combined kernels nearly always have better prediction performance than TPPK-combined kernels. In addition, our results suggest that the information of PPI and domain is more meaningful and promising than subcellular localization and phylogenetic profile on this problem. Furthermore, we could give a conclusion that the information of subcellular localization has nearly no influence on prediction of heterodimers.

An interesting perspective for future research is to design a new kernel based on the neighboring topological structure and weight-labeled edge information, or extract the useful sequence information of protein complexes by deep learning to solve this problem.

## Abbreviations

CMC: Clustering-based on maximal cliques
C-SVC: C-Support vector classification
GO: Gene ontology
LCPH: Literature-curated physical interaction
LLS: Log-likelihood score
MCL: Markov CLuster
MCODE: Molecular complex detection
MLPK: Metric learning pairwise kernel
NWE: Node-weighted expansion
PCP: Protein complex prediction
PPI: Protein-protein interaction
RNSC: Restricted neighborhood search clustering
RRW: Repeated random walks
SVM: Support vector machine
TAP: Tandem affinity purification
TPPK: Tensor product pairwise kernel
WI-PHI: Weighted yeast interactive enriched for direct PHysical interactions
